# Acute limb ischemia in an adolescent with COVID-19 and systemic scleroderma: a case report

**DOI:** 10.1186/s12887-022-03761-w

**Published:** 2022-12-28

**Authors:** Mark Jason D. C. Milan, Leonila F. Dans, Vanessa Maria F. Torres-Ticzon

**Affiliations:** 1 Department of Pediatrics, College of Medicine and Philippine General Hospital, University of the Philippines, Manila, Philippines; 2 Division of Pediatric Rheumatology, Department of Pediatrics, College of Medicine and Philippine, General Hospital, University of the Philippines, Manila, Philippines; 3 Division of Adolescent Medicine, Department of Pediatrics, College of Medicine and Philippine, General Hospital, University of the Philippines, Manila, Philippines

**Keywords:** COVID-19, SARS-CoV-2, Scleroderma

## Abstract

**Background:**

Juvenile Scleroderma is a rare autoimmune disease of the connective tissue. Its concurrence with COVID-19 can lead to limb ischemia as both disease entities are pro-inflammatory and pro-thrombotic. To date, there is no case report describing the symptomatology and course of disease in patients with juvenile Scleroderma and COVID-19.

**Case presentation:**

An adolescent with acute limb ischemia presented with a history of generalized hypo-and-hyperpigmented skin lesions and mild, non-productive cough. She tested positive for SARS-CoV-2 on nasopharyngeal swab RT-PCR. Further work-up revealed elevated anti-phospholipid antibodies, anti-nuclear antibody, and D-dimer; low Protein S activity; and evidence of peripheral arterial disease on imaging studies. She was started on peripheral vasodilators, Methotrexate, and anticoagulation. Close monitoring of the affected limbs and other organs involved was done. Control of limb ischemia was achieved after 4 months of regular Cyclophosphamide infusion. Continued multi-disciplinary care was ensured for this patient.

**Conclusion:**

There is evolving knowledge about the interplay of COVID-19 hyperinflammatory state and rheumatologic disorders. COVID-19 is thought to exacerbate cutaneous manifestations of autoimmune disorders via antigen protein mimicry and cytokine imbalance. Moreover, COVID-19 is characterized by complex hematopathologic processes that put a patient in a hypercoagulable state. Elevated D-dimer can be seen in both COVID-19 and systemic sclerosis owing to their pro-thrombotic sequela. There is scarcity of data on the association of Protein S activity with COVID-19 and systemic sclerosis. More studies need to be carried out to ultimately arrive at a consensus on thrombosis prophylaxis for patients with Scleroderma and COVID-19.

## Background

Coronavirus disease 2019 (COVID-19) is a novel disease caused by the SARS-CoV-2 virus that originated in Wuhan, China. By the end of October 2022, the reported confirmed cases of COVID-19 reached 627 M, with about 6.5 M deaths globally [1]. COVID-19 has no pathognomonic clinical features, but global data show that the most commonly reported signs and symptoms in the pediatric population are fever and respiratory symptoms [2–5]. Given this set of non-specific symptoms, COVID-19 is difficult to differentiate from other respiratory diseases, especially during the initial phase of the disease. Despite SARS-CoV-2 being a respiratory virus, it can also affect the other systems of the body. Of particular note is the hemostatic pathway. Inflammatory mediators implicated during SARS-CoV-2 infection have been postulated to cause endothelial damage and hypercoagulability, eventually leading to microthrombi and limb ischemia [6]. Although a rare complication of COVID-19, acute limb ischemia (ALI) can present in its worse form among patients with systemic Scleroderma and concomitant COVID-19 infection. In this study, we report a case of a pediatric patient with Scleroderma who contracted COVID-19 and developed ALI. This paper describes the symptomatology of COVID-19 and Scleroderma overlap, the similarities in their pathophysiology, and the approach to management.

## Case presentation

**Fig. 1:**
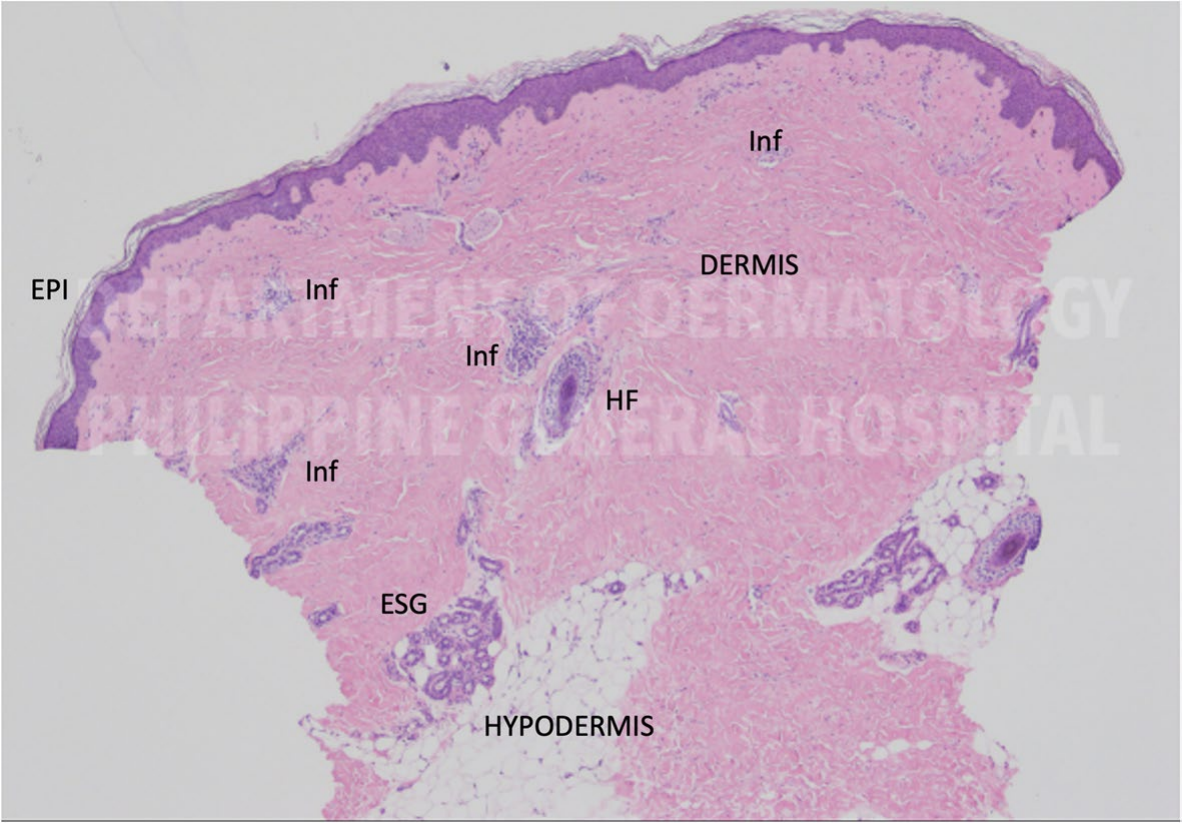
Histologic cross-section of skin biopsy showing slightly thinned epidermis with reduced skin adnexal structures. Notable thickened and hyalinized dermis with scattered cellular infiltrates (Inf). Hair Follicle (HF), eccrine sweat glands (ESG)

An adolescent female with acute limb ischemia tested positive for COVID-19 at the emergency room. Her history of present illness started 1 year and 8 months prior to admission when she presented with generalized erythematous skin patches that progressed to generalized hyper-and-hypopigmentation (salt-and-pepper pattern). She had intermittent arthralgia, weight loss, and cyanosis of the distal digits when exposed to cold temperatures. The cyanotic digits were tender but eventually became erythematous and painless. She also had persistent skin dryness, which prompted a consult with a dermatologist. Systemic sclerosis was initially suspected, and a skin biopsy revealed sclerosing dermatosis (Fig. 1). She was seen in a Rheumatology outpatient clinic for evaluation of systemic Scleroderma but was eventually lost to follow-up. In the interval, she was noted with Raynaud phenomenon, which, 1-week prior to admission, progressed to ischemia of the distal digits of the right hand and cyanosis of the fingers and toes of the other extremities. She consulted back to the rheumatology clinic, where she was started on Sildenafil 1 mg/kg/dose two times a day and Amlodipine 0.4 mg/kg/dose once a day. The medications were taken with good compliance; however, there was still progression of ischemia of the distal extremities. This prompted a consult at the emergency department. The review of systems revealed a 1-week history of non-productive cough with no associated dyspnea. She has no known co-morbidities, previous surgeries, or prior hospital admissions. There was no similar condition in their family. There was also no known case of COVID-19 in the household.

**Fig. 2:**
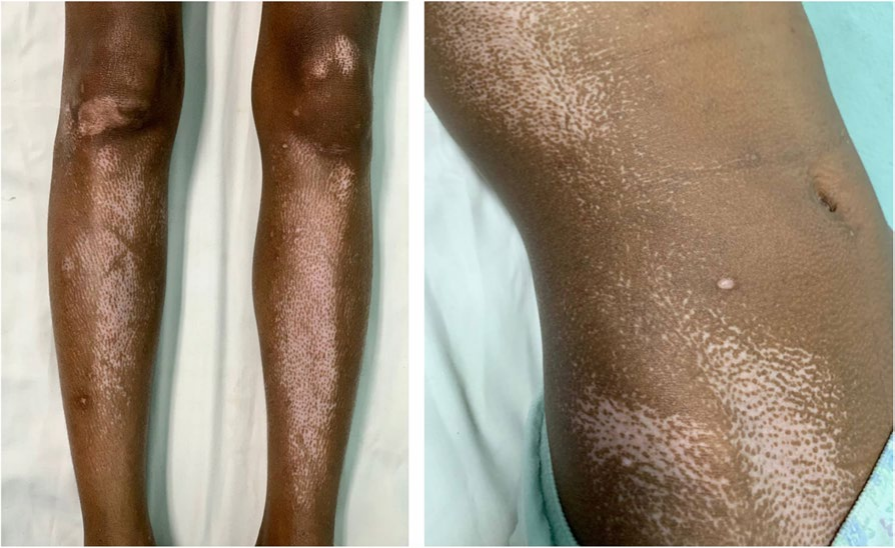
Generalized hyper-and-hypopigmentation of the skin of the anterior legs (Left photo) and lateral aspect of the abdomen (Right photo) in a patient with Systemic Scleroderma

**Fig. 3:**
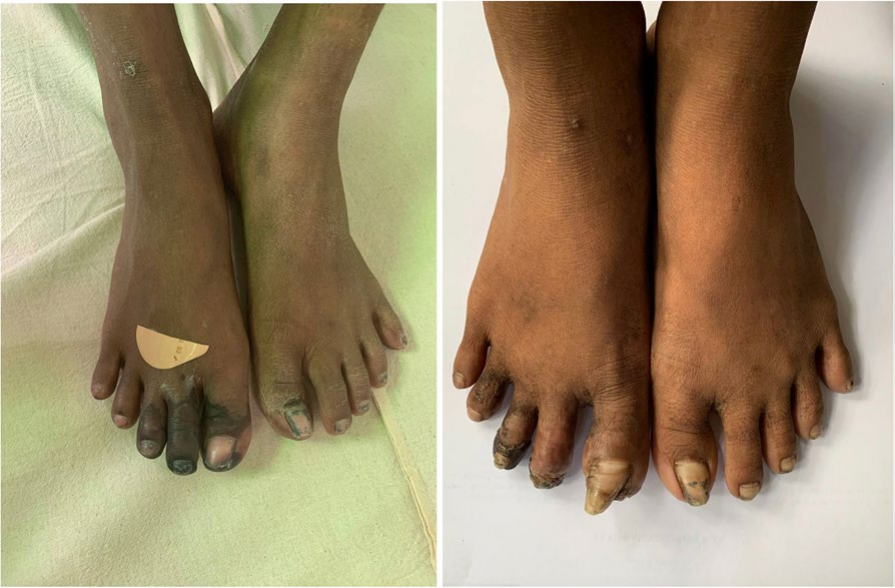
Comparison of right foot digital ischemia pre-Cyclophosphamide infusion (Left photo) and after 2nd cycle of Cyclophosphamide (Right photo)

She was brought to a tertiary government university hospital and designated COVID-19 referral center in Manila, Philippines. At the emergency department, she was seen awake, not in distress, but non-ambulatory due to tender bilateral toes. Vital signs were as follows: BP 102/64 mmHg, heart rate 111 bpm, respiratory rate 20 cpm, peripheral Oxygen saturation 98%, and temperature 36.5 °C. Her cardiovascular, chest, abdominal, and genitourinary examinations were unremarkable, but notable in the systemic examination was the generalized hyper-and-hypopigmentation of the skin appearing in salt-and-pepper pattern (Fig. 2). She had multiple dental caries but had no feeding or oromotor deficits. She had violaceous discoloration and sensory deficits of the distal phalanges of bilateral hands and feet (Fig. 3). The patient did a subjective report of sensory function during the neurologic physical exam. She reported a 50% sensory deficit at the 3rd, 4th, and 5th distal phalanges of the left hand; 2nd distal phalanx of the right hand; 1st distal phalanx of the left foot; and all distal phalanges of the right foot. There was absent sensation over the 3rd and 4th distal phalanges of the right hand.

Routine COVID-19 point-of-care testing was done using SARS-CoV-2 reverse transcriptase polymerase chain reaction (RT-PCR), and she tested positive. She was subsequently admitted for limb-salvaging interventions. Radiographic imaging of the bilateral hands and feet was negative for joint space or osseous abnormality. Doppler studies were done for the ischemic limbs and showed triphasic bilateral radial, ulnar, and brachial pulses. No surgical intervention was contemplated for the patient at the time of admission, and maximization of medical management was prioritized. Iloprost was the initial drug of choice for ischemic ulcers but was not started due to its unavailability in the institution. She was then started on Nifedipine 0.4 mg/kg/dose every 6 hours, Sildenafil 2 mg/kg/dose three times a day, and Nitroglycerin patch over the affected digits. Prednisone and Naproxen sodium were started for anti-inflammation and analgesia, respectively but were eventually discontinued.

The concurrence of COVID-19 with juvenile systemic Scleroderma was postulated to have triggered the limb ischemia. Additional tests done for this patient revealed normal PT-INR, elevated D-dimer levels, normal protein C, low protein S, and normal Fibrinogen levels. To date, there is no international protocol in the initiation of venous thromboembolism prophylaxis for patients with systemic Scleroderma with concomitant COVID-19. This patient, however, was started on Enoxaparin 0.5 mg/kg/dose subcutaneously every 12 hours and was carefully monitored for signs of bleeding and heparin-induced thrombocytopenia. While at the COVID ward, she had worsening sensory deficits on the 4th and 5th digits of the left hand but had no progression of the ischemia. She also had no new-onset pain or pallor in the uninvolved digits. While awaiting for the peripheral angiography, she underwent arterial doppler studies with digit-brachial index measurement. The examination revealed monophasic to biphasic signals at the distal bilateral radial and ulnar arteries and monophasic signals of the left anterior tibial artery and bilateral peroneal arteries; which suggest peripheral arterial disease of the bilateral upper and lower extremities.

After finishing 14 days of isolation at the COVID ward, she was subsequently transferred to the non-COVID ward for the continuation of management. She was started on Methotrexate 0.3 mg/kg/dose twice a day weekly and Folic acid 0.2 mg/kg/dose for the diffuse cutaneous disease. Because she had persistent and progressive skin involvement, she was started on Cyclophosphamide 500 mg per body surface area (BSA) and Uromitexan 40% of the cyclophosphamide dose. No hematuria was noted during the course of therapy. She was seen by the orthopedics service and was advised to undergo left small distal interphalangeal joint disarticulation; however, the parents did not consent to it. Medical management was then continued, and no untoward reactions were noted while she was on Methotrexate. Peripheral angiography was subsequently done, revealing complete occlusion of the proximal right dorsalis pedis artery, diminutive left ulnar and interosseous arteries, and diminutive left 4th and 5th digital arteries. Vasodilators were continued, and she was noted to have improvement of the distal extremities after 4 weeks. No progression of ischemia was noted in the interval, and she was discharged with regular follow-up for Cyclophosphamide infusion on outpatient basis.

Our patient was managed in a tertiary COVID-19 referral institution. She was classified as having mild COVID-19 because of a history of non-productive cough but with unremarkable chest radiograph. Immunologic work up for systemic Scleroderma was done, revealing positive Anti-nuclear antibody and elevated Anti-phospholipid antibodies. Detection of Systemic sclerosis-selective autoantibodies was not done due to its unavailability in the institution. The following adjunctive tests were also done: Protein C (80%, normal value: 70-130%), Protein S (37%, normal value: 72-106%), and Erythrocyte sedimentation rate (4 mm/hr., normal value: 0-15 mm/hr). D-dimer was elevated at 0.79 μg/mL (prolonged for age, normal value: 0.16-0.39 μg/mL). Low Protein S activity and elevated D-dimer were attributed to the pro-thrombotic state of the patient.

Investigations for vascular pathology using Arterial Doppler with segmental pressures and waveform studies revealed peripheral arterial disease. Four-extremity angiogram showed luminal narrowing of distal arteries only on the bilateral upper extremities.

To date, there is no established guideline for the initiation of anti-thromboembolic prophylaxis for patients with Scleroderma and concomitant COVID-19 infection. However, because of the predominantly hypercoagulable state of COVID-19 patients on top of the vascular pathology in Scleroderma, it was deemed prudent to start the patient on Enoxaparin. For the Scleroderma-related Raynaud phenomenon, the goals were to increase comfort and prevent secondary ischemic sequelae, which can be achieved using arteriolar vasodilators [7, 8]. The patient was maintained on Nifedipine 10 mg/tab every 8 hours, Sildenafil 25 mg/tab every 12 hours, and Nitroglycerin patch. Central to the management of Raynaud phenomenon is avoidance of the precipitating circumstances such as cold and stress, and this was emphasized to the patient [7, 8]. Severe digital ulcers are often addressed with Prostacyclin, but due to its unavailability, the patient was started on Bosentan instead [9, 10]. The standard therapy to address the diffuse skin involvement in systemic Scleroderma is Methotrexate, with which the patient had good compliance [7, 8]. Due to progressive skin thickening and persistence of cutaneous lesions, she was started on Cyclophosphamide with subsequent infusions done on outpatient basis.

On follow-up, the patient still experienced Raynaud phenomenon, but there was no progression of the digital ulcers. She has had a total of 5 cycles of Cyclophosphamide infusion, and her previously cyanotic but non-ischemic digits have significantly improved. The adolescent medicine, child psychiatry, rehabilitation medicine, and gastroenterology services were actively managing this patient to help her cope with the burden of chronic autoimmune disease. Adherence to treatment was ensured by providing regular online, and face-to-face clinic consults, providing free medicines and diagnostics through the medical social services, and involving the family in the patient’s care.

## Discussion and conclusions

Juvenile systemic Scleroderma is an autoimmune disease characterized by vasospasms that lead to digital ischemia and ulcers. Moreover, it is thought to cause vascular endothelial injury that may increase the risk of thrombosis [11, 12]. The criteria for Systemic sclerosis fulfilled by this patient were presence of skin thickening proximal to the metacarpal phalangeal (MCP) joints, Raynaud Phenomenon, arthritis, sclerodactyly, and presence of Anti-nuclear antibodies on serology. She had elevated D-dimers on routine COVID-19 work-up. Marie and colleagues in 2008 explained that elevated levels of D-dimer are associated with macrovascular impairment that leads to peripheral ischemia in Systemic sclerosis [13], while its elevation has been observed to correlate with illness severity among COVID-19 patients [12, 14]. Endothelial cell changes in COVID-19 and Scleroderma are thought to result in increased vascular permeability, elevated D-dimer and PAI-1 levels, and expression of adhesion molecules. Moreover, both disease entities are characterized by increased platelet activation, coagulation, microthrombosis, and fibrinolysis [15]. Immune activation and the reduced expression of Angiotensin Converting Enzyme II (ACE II) are common in both disease entities, especially during the early phase [15]. In contrast to chronic autoimmune vascular insult in Scleroderma, COVID-19’s vascular pathology is a result of inflammation from acute viral infection. Nevertheless, both disease processes can ultimately result in tissue hypoperfusion owing to vascular fibrosis in Scleroderma and a pro-thrombotic state in COVID-19 [15]. These pathophysiologic mechanisms may help guide clinicians in preventing the progression of vasculopathy and limb ischemia in patients with Scleroderma and COVID-19. The association of Protein S with COVID-related thrombosis has not yet been well-elucidated. Because COVID-19 is a relatively new disease entity, more data has yet to be generated about its impact on the pattern of symptomatology and the course of other diseases. To date, there is limited data on the effect of COVID-19 on the natural course of rheumatologic diseases. However, similarities between some rheumatologic disorders and COVID-19 sequelae have been observed. Of particular note is an important life-threatening post-COVID hyper-inflammatory condition, formally known as multisystem inflammatory syndrome in children (MIS-C), which presents with strikingly similar features to Kawasaki disease and macrophage activation syndrome [16]. The similarities were linked to the hyperinflammatory state of COVID-19 [16]. This hyperinflammatory state consequently puts patients with pediatric inflammatory rheumatologic diseases at an increased risk of hospitalization and the development of symptomatic COVID-19 [14]. The American College of Rheumatology (ACR) recommends temporarily delaying the provision of immunosuppressants and continuing the use of lowest possible dose of steroids to control the underlying disease in children with rheumatologic disorders and COVID-19 [17]. We present this case to demonstrate that COVID-19 can aggravate limb ischemia in patients with Systemic Scleroderma. This report adds to the evolving knowledge of the interaction between COVID-19 and hyperinflammation in a rheumatologic disorder. More studies, however, need to be carried out in order to demonstrate associations objectively and ultimately arrive at a consensus on thrombosis prophylaxis for patients with Scleroderma and COVID-19.

## Data Availability

Data sharing is not applicable to this article as no datasets were generated or analyzed during the current study.

## Abbreviations

aPTT: Activated Partial Thromboplastin Time
BSA: Body surface area
COVID-19: Coronavirus disease 2019
PT-INR: Prothrombin Time – International Normalized Ratio
RT-PCR: Reverse Transcription – Polymerase Chain Reaction
